# Causal relationships between plasma lipids and sepsis: A Mendelian randomization study

**DOI:** 10.1097/MD.0000000000036288

**Published:** 2023-12-08

**Authors:** Jing Chen, Wei Chen, Lin Wu, Rong Hui Wang, Jun Jun Xiang, Fu Kui Zheng, Qiao Ming Huang

**Affiliations:** a The First Affiliated Hospital of Guangxi University of Traditional Chinese Medicine, Nanning, China; b Guangxi University of Chinese Medicine, Nanning, China.

**Keywords:** high-density lipoprotein cholesterol, low-density lipoprotein cholesterol, Mendelian randomization, sepsis, triglycerides

## Abstract

Although observational studies have indicated that plasma lipids are associated with an increased risk of sepsis, due to confounders and reverse causality, the causal relationship remains unclear. This study was designed to assess the causal effects of plasma lipid levels on sepsis. We used a 2-sample Mendelian randomization (MR) method to evaluate the causal effect of plasma lipids on sepsis. MR analysis employs methods such as inverse variance weighted, MR-Egger regression, weighted median regression (WME), simple mode and weighted mode. The inverse variance weighted (IVW) method was predominantly utilized to assess causality. Heterogeneity was affirmed by Cochran Q test, while pleiotropy was corroborated by MR-Egger regression analysis. The robustness and reliability of the results were demonstrated through “leave-one-out” sensitivity analysis. Instrumental variables included 226 single-nucleotide polymorphisms (SNPs), comprising of 7 for triglyceride (TG), 169 for high-density lipoprotein cholesterol (HDL-C), and 50 for low-density lipoprotein cholesterol (LDL-C). The risk of sepsis appeared to increase with rising LDL-C levels, as indicated by the inverse variance weighted analysis (OR 1.11, 95% CI from0.99 to1.24, *P* = 0.068). However, no causality existed between LDL-C, HDL-C, TG and sepsis. Two-sample MR analysis indicated that increased LDL-C level is a risk factor for sepsis, while TG and HDL-C levels have protective effects against sepsis. However, no significant causal relationship was found between TG, HDL-C, and LDL-C levels and sepsis.

## 1. Introduction

Sepsis is a severe condition that can lead to life-threatening organ dysfunction. It is fundamentally characterized by inflammation and immune dysregulation provoked by infection,^[1]^ with prominent features such as long-term disease onset, high mortality rate in late stages, and chronic severity.^[2]^ In 2017, approximately 48.9 million people worldwide were affected by sepsis. Sepsis poses a significant challenge to global health.^[3]^ Despite significant advancements in medical technology, The incidence and mortality rates attributed to sepsis continue to increase, and survivors of sepsis commonly endure a diminished quality of life. Notably, the mortality risk remains elevated,^[4]^ presenting substantial socio-economic costs.^[5,6]^ Several patients may endure complications, encompassing physiological, psychological, and cognitive functional impairments.^[7]^ The treatment cost for sepsis is considered the highest among all diseases in Intensive Care Units.^[8]^ Currently, there is no specific treatment available for sepsis. Consequently, it is crucial to identify risk factors that influence sepsis onset and prognosis.

Plasma lipids include triglyceride (TG), total cholesterol (TC), low-density lipoprotein cholesterol (LDL-C), and high-density lipoprotein cholesterol (HDL-C). Triglyceride represents the largest energy storage in the human body and accumulate in the cytoplasm of fat cells.^[9]^ In the early stages of sepsis, the level of fat breakdown in adipose tissue is increased,^[10,11]^ leading to elevated levels of triglycerides, with septic patients showing plasma triglyceride concentrations approximately 3 times higher than that in healthy individuals.^[12]^ Cholesterol is a major lipid in the human body, and prior research has illustrated an association between low serum cholesterol levels and numerous disorders, including heart failure, ischemic stroke, cancer, sepsis, and myocardial infarction.^[13–15]^Although cholesterol is a risk factor for cardiovascular and cerebrovascular events, it offers a protective effect in sepsis.^[16]^ LDL-C can promote the clearance of bacterial toxins,^[17]^whereas HDL-C can bind to bacterial toxins, preventing the release of inflammatory factors.^[16,18]^ There is a decrease in the levels of HDL-C that can predict the risk of infection and worsening outcomes in sepsis.^[19]^Reduced lipoprotein cholesterol levels are associated with increased organ failure and mortality rates in septic patients.^[20–22]^ Cardiovascular risks may be provoked by abnormal blood lipid levels, but the causal effect of lipids on sepsis is not yet clear. Therefore, studies utilizing Mendelian randomization (MR) are in progress to investigate the potential causal relationship between plasma lipid levels and sepsis.

MR, originally introduced by Katan (1986), employs genetic variations as instrumental variables to evaluate the causal relationship between exposure and outcomes, thereby mitigating confounding factors and reverse causality.^[23]^ This methodology parallels the conventional randomized controlled trials but is designed to mitigate the inherent confounding elements within randomized controlled trials.^[24]^ In this study, we used a 2-sample MR method to analyze the causal relationship between plasma lipids and sepsis.

## 2. Materials and methods

### 2.1. Study design

**Figure 1.**
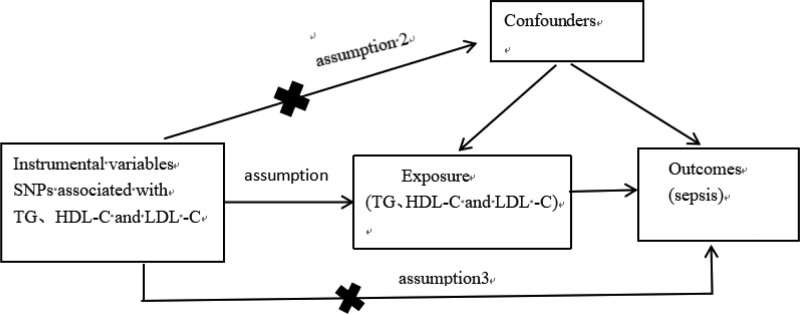
The schematic diagram of Mendelian randomization.

**Figure 2.**
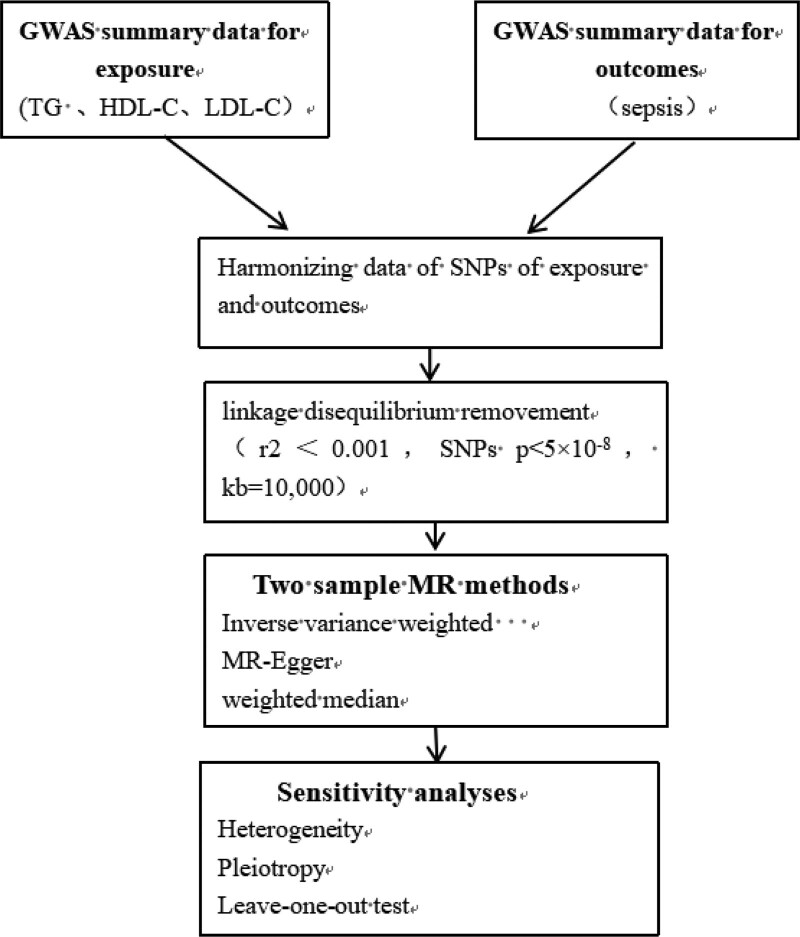
The flowchart of the study.

MR, grounded in the principle that varying genotypes dictate distinct intermediate phenotypes representing individual exposures, posits that the correlation between genotype and disease mirrors the impact of the exposure on the disease.^[25]^ As shown in Figure 1, Single nucleotide polymorphisms (SNPs) functioned as instrumental variables (IVs) to infer the association between exposure and outcome. An MR analysis must satisfy 3 fundamental assumptions^[26]^: relevance Assumption: SNPs must have a strong association with exposure, exclusion Restriction Assumption: The SNPs should not be associated with the outcome, and independence assumption: SNPs were not associated with any confounding factors. The flowchart of the study is shown in Figure 2.

### 2.2. Data sources

Genome-Wide Association Studies (GWAS) data for TG (GWAS ID: ieu-a-302), HDL-C (GWAS ID: ieu-b-109), LDL-C (GWAS ID: ieu-b-110), and sepsis (GWAS ID: ieu-b-4980) were retrieved from the IEU OpenGWAS database. The corresponding summary information is presented in Table 1. The ethical approval in our study was not required, because of available data in public GWAS data set (https://gwas.mrcieu.ac.uk).

**Table 1:** Summary of the GWAS included in this 2-sample MR study.

Variable	ID	Sample size	Number of SNPS	Consortuim	Population	Sex	Yr
TG	ieu-a-302	177,861	2439,433	GLGC	Mixed	Males and Females	2013
HDL-C	ieu-b-109	403,943	12,321,875	UK Biobank	European	Males and Females	2020
LDL-C	ieu-b-110	440,546	12,321,875	UK Biobank	European	Males and Females	2020
Sepsis	ieu-b-4980	486,484	12,243,539	UK Biobank	European	Males and Females	2021

GWAS = Genome-Wide Association Studies, MR = Mendelian randomization, HDL-C = high-density lipoprotein cholesterol, LDL-C = low-density lipoprotein cholesterol, SNPs = single-nucleotide polymorphisms, TG = triglyceride.

### 2.3. Selection of instrumental variables

We selected instrumental variables (IVs) in the following manner: we chose significant SNPs (*P* < 5.0 × 10^−8^) as instrumental variables. The F-statistics were computed using the subsequent equation to evaluate the potency of each instrumental variable (IV). To mitigate the bias induced by ineffective instrumental variables (IVs), those with an F-statistic <10 were omitted.^[27]^ The *F* statistics was formulated as $F=\frac{R^{2}\left(n-1-K\right)}{\left(1-R^{2}\right)K}$, “R2” signifies the proportion of variation accounted for by the SNPs present in the exposure, whereas “n” illustrates the sample size and “K” indicates the total number of Instrumental Variables (IVs). and we set the imbalance ratio r^2^ < 0.001, and set kb equal to 10,000 to minimize the interference from linkage disequilibrium.^[28]^ We excluded SNPs related to confounding factors, including body mass index, chronic liver disease, infection, andage, etc, using the Phenoscanner database (http://www.phenoscanner.medschl.cam.ac.uk/).^[29]^

### 2.4. Statistical analysis for mendelian randomization

MR analysis employs methods such as inverse variance weighted, MR-Egger regression,^[30]^ weighted median regression,^[31]^ simple mode and weighted mode. The inverse variance weighted (IVW) method was predominantly utilized to assess causality. The Inverse Variance Weighting (IVW) method, adopting multiplication effects, is utilized to ascertain the causal relationship between exposure and outcomes. Predicated on weighted least squares regression, the Inverse Variance Weighting (IVW) method employs genetic variation as an instrumental variable to gauge the resultant influence of exposure factors on given outcomes. Contrasting with many regression models, the IVW regression does not incorporate an intercept term. Consequently, the final outcome manifests as the weighted average of the effect values attributed to all instrumental variables (IVs).by contrast, MR-Egger regression considers the existence of an intercept term. The Weighted Median Estimate represents the median of the distribution function, which is determined after arranging the SNP effect values based on their respective weights, allowing for the correct estimation of cause-effect relationships even when up to 50% of the instrumental variables are invalid^.[31]^ IVW was the primary analysis method in this study, with MR-Egger and the weighted median method used to supplement the IVW estimation. Heterogeneity was affirmed by Cochran Q test,^[32]^ while pleiotropy was corroborated by MR-Egger regression analysis. The robustness and reliability of the results were demonstrated through “leave-one-out” sensitivity analysis. There was no evidence of directional pleiotropy, as indicated by an Egger-intercept from the linear regression approximating zero, and undetectable horizontal pleiotropy of the instrumental variables (IVs), as supported by a *P*>.05 in the MR-PRESSO global test. Thereby, we could deem the exclusivity assumption as valid. All the above methods were implemented using the TwoSampleMR package in R 4.2.2. Statistically significant difference was considered at *P* < .05.

## 3. Results

### 3.1. Instrumental variables

In the study, we identified 226 independent (r^2^ < 0.001) and significantly correlated SNPs (*P* < 5 × 10^−8^) to serve as instrumental variables, excluding the following SNPs: rs10119644, rs1125873, rs133015, rs407133, rs990619.Among them, there are 7 triglycerides (TG), 169 high-density lipoproteins (HDL-C), and 50 low-density lipoproteins (LDL-C). The distribution range of the F statistic for a single SNP was 29.86 to 5569.87, This implies that the causal association is less susceptible to distortions caused by weak instrumental variable bias. Detailed results are provided in Table 2.

**Table 2:** The Mendelian randomization analysis results with regard to causal efect of TG,HDL-C and LDL-C on sepsis.

exposure	SNPs (n)	IVW				WME			MR-Egger	
		OR (95%CI)	Q (*P*-value)	*P* value	OR (95%CI)		*P* value	OR (95%CI)		*P* value
TG	7	0.96 (0.82, 1.12)	5.292 (0.507)	.605	0.94 (0.79, 1.13)		.395	0.90 (0.71, 1.15)		.438
HDL-C	169	0.94 (0.86,1.03)	222.769 (0.003)	.198	0.97 (0.8, 1.11)		.971	1.04 (0.90, 1.19)		.617
LDL-C	50	1.11 (0.99, 1.24)	56.380 (0.218)	.068	1.20 (1.0, 1.38)		.082	1.18 (0.9, 1.39)		.0648

HDL-C = high-density lipoprotein cholesterol, IVW = the inverse variance weighted, LDL-C = low-density lipoprotein cholesterol, MR = Mendelian randomization, OR = odds ratio, TG = triglyceride, WME = weighted median regression.

### 3.2. Two-sample MR analysis

**Figure 3.**
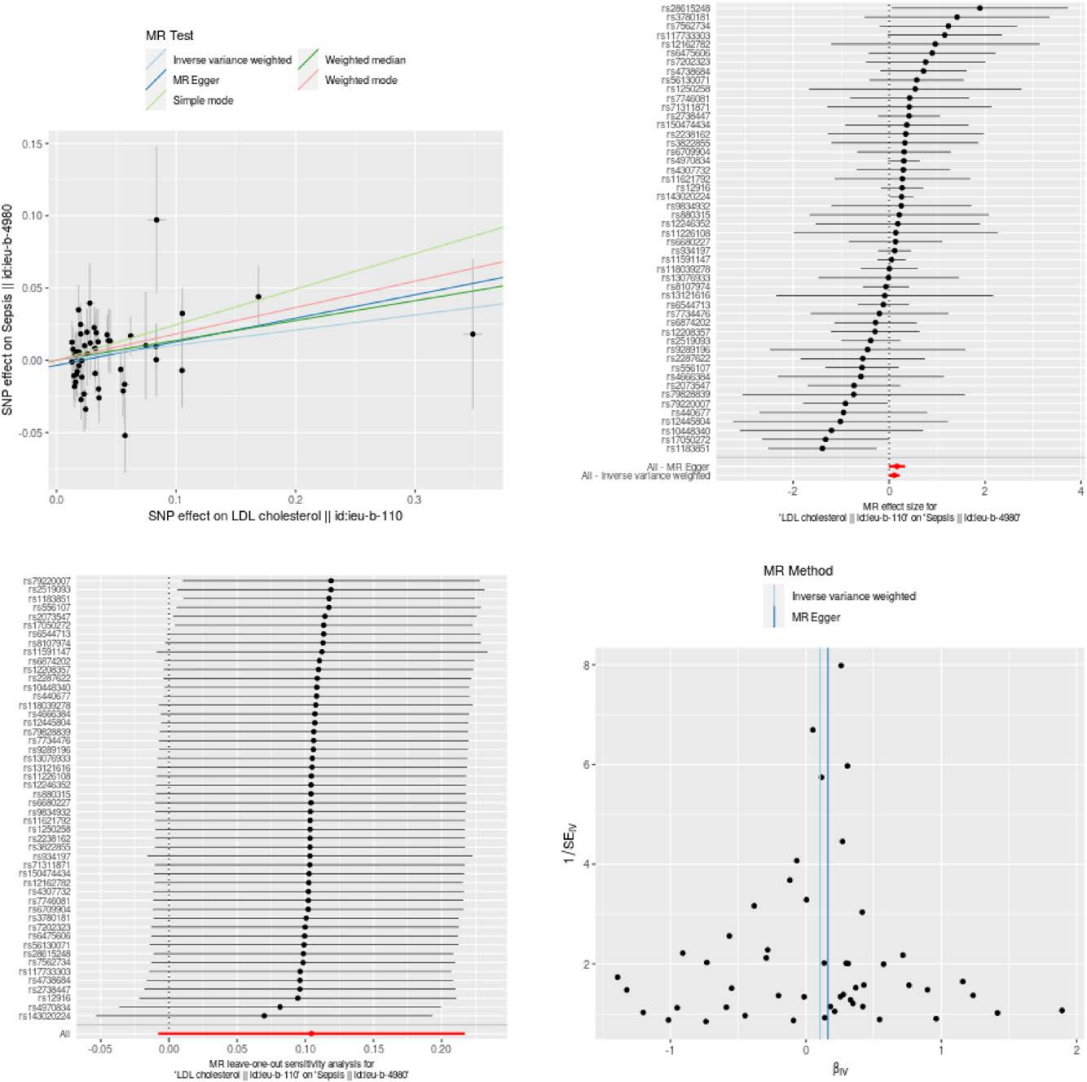
The MR results of LDL-C on sepsis. LDL-C = low-density lipoprotein cholesterol, polymorphisms.

**Figure 4.**
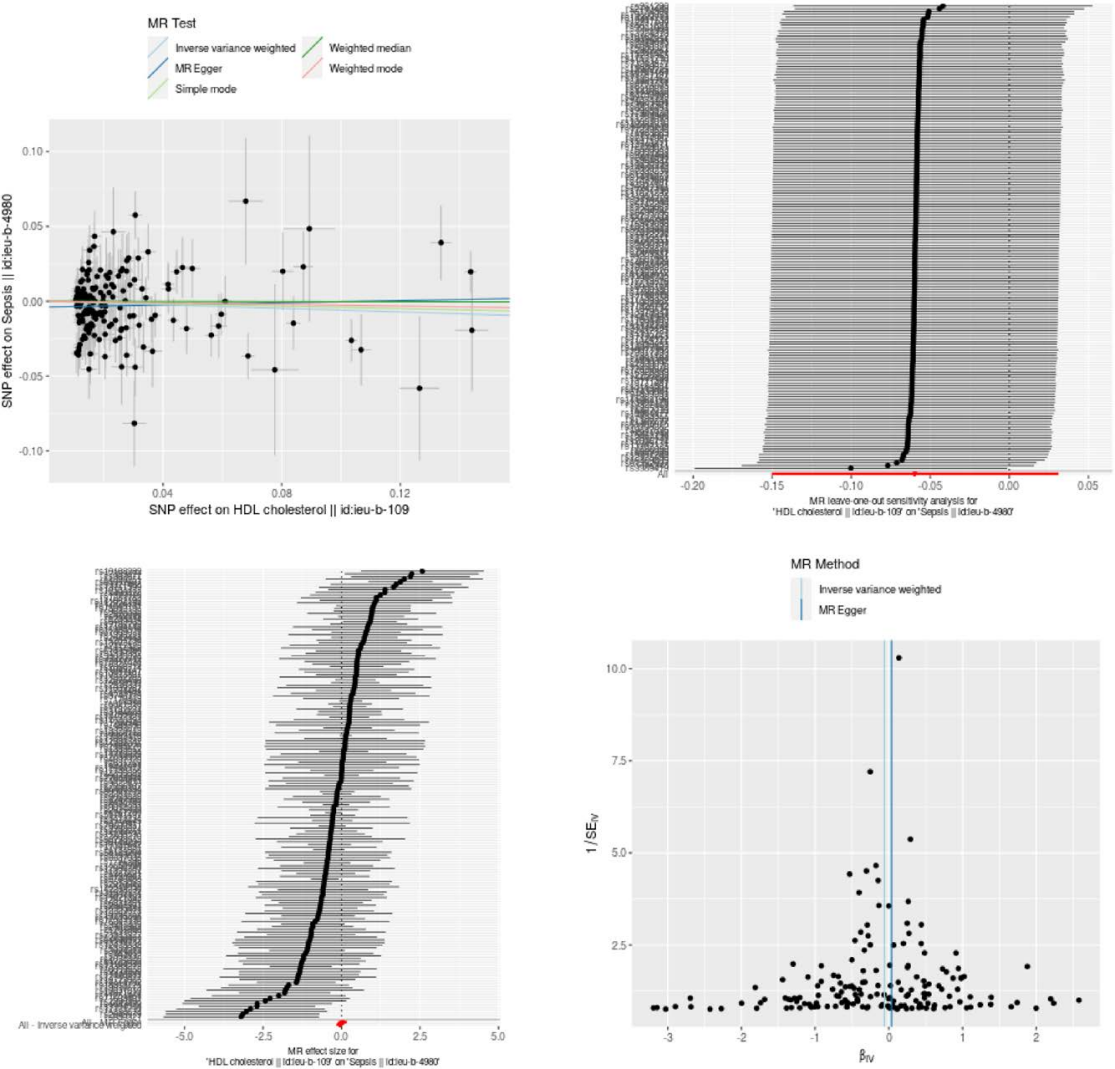
The MR results of HDL-C on sepsis. HDL-C = high-density lipoprotein cholesterol, MR = Mendelian randomization.

**Figure 5.**
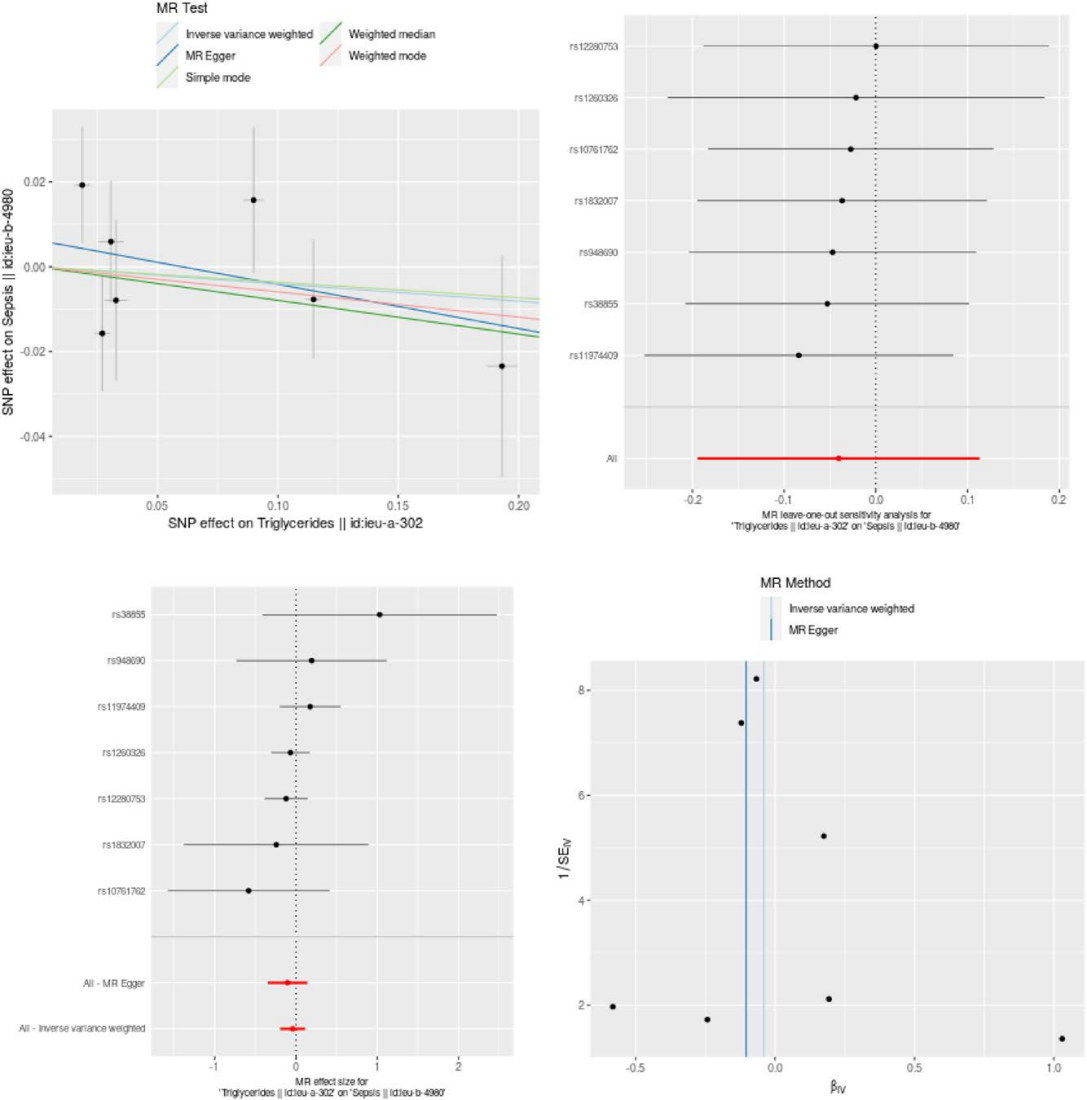
The MR results of TG on sepsis.

As shown in Figures 3, 4, and 5, LDL-C was a risk factor of sepsis (OR: 1.11;95% CI: from 0.99 to1.24; *P* = .068) with no heterogeneity (*Q* = 56.380, *P* = .218), while TG and HDL-C levels have protective effects against sepsis. However, no significant causal relationship was found between TG, HDL-C, and LDL-C levels and sepsis in the IVW result respectively (OR: 0.96;95% CI: from 0.82 to1.12; *P* = 0.605), (OR: 0.94;95% CI: from 0.86 to1.03; *P* = .198) and (OR: 1.11;95% CI: from 0.99 to1.24; *P* = .068).

### 3.3. Sensitivity analysis

The F-statistic for all instrumental variables (IVs) exceeded 10, suggesting a higher precision and accuracy in our estimation of the causal association between

TG, HDL-C, LDL-C and sepsis. As shown in Table 2, no directional pleiotropy and horizontal pleiotropy was found no directional pleiotropy and horizontal pleiotropy was found for the analyze of TG, HDL-C and LDL-C. In the funnel plot, with each point representing the causal association effect when employing a single SNP as an IV, revealed a symmetric distribution. This symmetry suggests that causal associations are less prone to potential bias. As can be seen from the funnel plot, all the included SNPs are symmetrical, indicating that the estimation of causal effects using SNPs as IVs experiences relatively small potential impacts. The results of the pleiotropy analysis are presented in a scatter plot. The results indicated that there was no horizontal pleiotropy (TG, egger_intercept = 0.0062, *P* = .536; HDL-C, egger_intercept = −0.0038, *P* = .0793; LDL-C, egger_intercept = −0.0035, *P* = .3692), as shown in Figures 3, 4, and 5.

## 4. Discussion

In this study, a 2-sample MR approach was utilized to investigate the causal relationship between plasma lipids and sepsis, using GWAS data. The results indicated that LDL-C is a risk factor for sepsis, while TG and HDL-C are protective factors against sepsis.

In a prospective cohort study of 171 sepsis patients, total cholesterol, LDL-C and HDL-C levels were significantly decreased in 113 bacterial culture positive sepsis cases (TC, *P* < .001; LDL-C, *P* < .001; HDL-C, *P* = .011). Moreover, cholesterol levels differed among sepsis patients with different types of infections, with gram-negative bacteremia patients having significantly lower cholesterol levels than Gram-positive bacteremia patients.^[33]^ A retrospective study found that among 4512 sepsis patients, both the low-cholesterol group (cholesterol < 120 mg/dL) and the high-cholesterol group (cholesterol > 200 mg/dL) had a higher risk of death at 28 days than the normal cholesterol group (cholesterol 120–200 mg/dL). This suggests that the cholesterol concentration can be used as a prognostic factor for sepsis or septic shock.^[14,34]^ Phase I clinical studies have reported that stable cholesterol levels in early sepsis can be managed using fish oil-infused Lipid Emulsions. However, whether this type of exogenous lipid emulsion can alleviate complications such as organ failure and mortality rate. still needs further research.^[35]^ HDL-C concentrations are positively associated with the alleviation of sepsis, suggesting a potential therapeutic role for HDL-C. Recombinant HDL treatment can protect animal models of sepsis from organ damage and improve survival rates.^[36]^ HDL regulates the inflammatory pathway in sepsis via the transcription regulatory factor ATF3 and can also protect endothelial cells.^[37]^ Lower HDL-C levels are associated with early onset of sepsis, increased risk of organ failures, and higher sepsis related mortality rates.^[38,39]^ Genetic analysis of HDL-C PRS, CETP PRS, and rs1800777 demonstrated a strong association between clinically measured HDL-C levels and sepsis risk. However, this was not correlated with the risk of sepsis or sepsis-related outcomes, suggesting that such beneficial clinical associations could potentially be attributed to confounding factors.^[40]^ The results from this MR study are consistent with the aforementioned observational studies but were unable to prove a causal relationship between triglycerides, low-density lipoprotein, and high-density lipoprotein with sepsis.

In conclusion, this study found LDL-C to be a risk factor for sepsis, while HDL-C and TG acted as protective factors. There was no causal relationship between TG, LDL-C or HDL-C and sepsis. But plasma lipids may have an impact on the progression of sepsis. Extensive research findings have indicated a higher mortality risk amongst patients with reduced concentrations of Triglycerides, High Density Lipoprotein, Cholesterol, and Low Density Lipoprotein Cholesterol.^[39–41]^ It suggested that the mortality rate from sepsis may decrease through enhanced lipid clearance of pathogenic bacteria via Low Density Lipoprotein clearance.^[41]^ A converse correlation is evident between serum inflammatory markers and cholesterol concentration.^[39]^ The biological mechanism triggering hypocholesterolemia in sepsis remains unclear and may be connected to factors such as decreased fat intake, intestinal absorption and synthesis, augmented metabolic products, and toxin clearance in Intensive Care Unit (ICU) patients. Lower cholesterol levels can potentially have detrimental effects on immune cells. During a septic episode, pathogen lipids are encapsulated within lipoproteins while the production of low-density lipoprotein diminishes. This decrease could limit the buffering capacity of LDL-C against pathogen lipids, possibly eliciting sepsis-related clinical symptoms. Therefore, augmenting HDL-C and TG levels and reducing LDL-C may ameliorate symptoms in septic conditions.

Compared with previous observational studies, the use of MR methods in this research greatly minimized the influence of confounding and reverse causality, resulting in more convincing causal relationships. Of course, this study has certain limitations. First, all GWASs data comes from European or mixed populations. At present, there is a lack of sepsis data information for Asia and Africa in the database, so the research results are not applicable to all ethnic groups, and whether these results are consistent among other ethnicities such as Asians needs further investigation. Second, lipids may have a causal relationship with sepsis caused by specific pathogenic factors, which should be considered for a broader study of disease-related sepsis in the future. Third, due to the design of working variable parameters, some lipid indicators were excluded during the screening process, so not all lipid indicators were included in this study. The results of this study only present a causal relationship between triglycerides, low-density lipoprotein, and high-density lipoprotein and sepsis, while other lipid indicators may have a causal relationship with sepsis.

## Acknowledgments

We extend our appreciation to the IEU Open GWAS database (https://gwas.mrcieu.ac.uk/) for its invaluable contributions to our research.

## Author contributions

**Conceptualization:** Jing Chen.

**Data curation:** Wei Chen, Qiao Ming Huang, Jun Jun Xiang.

**Formal analysis:** Jing Chen, Qiao Ming Huang.

**Resources:** Wei Chen.

**Supervision:** Jing Chen.

**Writing – original draft:** Jing Chen, Fu Kui Zheng, Jun Jun Xiang.

**Writing – review & editing:** Lin Wu, Rong Hui Wang.

## References

[1] Singer M Deutschman CS Seymour CW. The third international consensus definitions for sepsis and septic shock (sepsis-3). JAMA. 2016;315:801–10. 26903338 10.1001/jama.2016.0287 PMC4968574

[2] Rhodes A Evans LE Alhazzani W. Surviving Sepsis Campaign: international guidelines for management of sepsis and septic shock: 2016. Intensive Care Med. 2017;43:304–77. 28101605 10.1007/s00134-017-4683-6

[3] Rudd KE Johnson SC Agesa KM. Global, regional, and national sepsis incidence and mortality, 1990-2017: analysis for the Global Burden of Disease Study. Lancet. 2020;395:200–11. 31954465 10.1016/S0140-6736(19)32989-7 PMC6970225

[4] Huang M Cai S Su J. The pathogenesis of sepsis and potential therapeutic targets. Int J Mol Sci . 2019;20:5376. 31671729 10.3390/ijms20215376 PMC6862039

[5] Gardner AK Ghita GL Wang Z. The development of chronic critical illness determines physical function, quality of life, and long-term survival among early survivors of sepsis in surgical ICUs. Crit Care Med. 2019;47:566–73. 30664526 10.1097/CCM.0000000000003655 PMC6422682

[6] Guirgis FW Brakenridge S Sutchu S. The long-term burden of severe sepsis and septic shock: Sepsis recidivism and organ dysfunction. J Trauma Acute Care Surg. 2016;81:525–32. 27398984 10.1097/TA.0000000000001135

[7] Gaieski DF Edwards JM Kallan MJ. Benchmarking the incidence and mortality of severe sepsis in the United States. Crit Care Med. 2013;41:1167–74. 23442987 10.1097/CCM.0b013e31827c09f8

[8] Rocheteau P Chatre L Briand D. Sepsis induces long-term metabolic and mitochondrial muscle stem cell dysfunction amenable by mesenchymal stem cell therapy. Nat Commun. 2015;6:10145. 26666572 10.1038/ncomms10145 PMC4682118

[9] Wasyluk W Zwolak A. Metabolic alterations in sepsis. J Clin Med. 2021;10:2412. 34072402 10.3390/jcm10112412 PMC8197843

[10] Ilias I Vassiliadi DA Theodorakopoulou M. Adipose tissue lipolysis and circulating lipids in acute and subacute critical illness: effects of shock and treatment. J Crit Care. 2014;29:1130.e5–9. 10.1016/j.jcrc.2014.06.003 25012960

[11] Rittig N Bach E Thomsen HH. Regulation of lipolysis and adipose tissue signaling during acute endotoxin-induced inflammation: a human randomized crossover trial. PLoS One. 2016;11:e0162167. 27627109 10.1371/journal.pone.0162167 PMC5023116

[12] Nogueira AC Kawabata V Biselli P. Changes in plasma free fatty acid levels in septic patients are associated with cardiac damage and reduction in heart rate variability. Shock. 2008;29:342–8. 18000476 10.1097/shk.0b013e31815abbc6

[13] Nordestgaard BG Chapman MJ Humphries SE. European Atherosclerosis Society Consensus Panel Familial hypercholesterolaemia is underdiagnosed and undertreated in the general population: guidance for clinicians to prevent coronary heart disease: consensus statement of the European Atherosclerosis Society. Eur Heart J. 2013;34:3478–90a. 23956253 10.1093/eurheartj/eht273 PMC3844152

[14] Yamano S Shimizu K Ogura H. Low total cholesterol and high total bilirubin are associated with prognosis in patients with prolonged sepsis. J Crit Care. 2016;31:36–40. 26596698 10.1016/j.jcrc.2015.09.033

[15] Zhao W An Z Hong Y. Low total cholesterol level is the independent predictor of poor outcomes in patients with acute ischemic stroke: a hospital-based prospective study. BMC Neurol. 2016;16:36. 26980573 10.1186/s12883-016-0561-z PMC4793701

[16] Catapano AL Pirillo A Bonacina F. HDL in innateand adaptive immunity. Cardiovasc Res. 2014;103:372–83. 24935428 10.1093/cvr/cvu150

[17] Boyd JH Fjell CD Russell JA. Increased plasma PCSK9 levels are associated with reduced endotoxin clearance and the development of acute organ failures during sepsis. J Innate Immun. 2016;8:211–20. 26756586 10.1159/000442976 PMC6738828

[18] Murphy AJ Woollard KJ Suhartoyo A. Neutrophil activation is attenuated by high-density lipoprotein and apolipoprotein A-I in in vitro and in vivo models of inflammation. Arterioscler Thromb Vasc Biol. 2011;31:1333–41. 21474825 10.1161/ATVBAHA.111.226258

[19] Madsen CM Varbo A Tybjærg-Hansen A. U-shaped relationship of HDL and risk of infectious disease: two prospective population-based cohort studies. Eur Heart J. 2018;39:1181–90. 29228167 10.1093/eurheartj/ehx665

[20] Guirgis FW Dodani S Leeuwenburgh C. HDL inflammatory index correlates with and predicts severity of organ failure in patients with sepsis and septic shock. PLoS One. 2018;13:e0203813. 30216360 10.1371/journal.pone.0203813 PMC6138388

[21] Chien YF Chen CY Hsu CL. Decreased serum level of lipoprotein cholesterol is a poor prognostic factor for patients with severe community-acquired pneumonia that required intensive care unit admission. J Crit Care. 2015;30:506–10. 25702844 10.1016/j.jcrc.2015.01.001

[22] Maile MD Sigakis MJ Stringer KA. Impact of the pre-illness lipid profile on sepsis mortality. J Crit Care. 2020;57:197–202. 32182565 10.1016/j.jcrc.2020.01.016 PMC7391410

[23] Katan MB. Apolipoprotein E isoforms, serum cholesterol, and cancer. Lancet. 1986;1:507–8. 10.1016/s0140-6736(86)92972-7 2869248

[24] Lawlor DA Harbord RM Sterne JA. Mendelian randomization: using genes as instruments for making causal inferences in epidemiology. Stat Med. 2008;27:1133–63. 17886233 10.1002/sim.3034

[25] Davey Smith G Hemani G. Mendelian randomization: genetic anchors for causal inference in epidemiological studies. Hum Mol Genet. 2014;23:R89–98. 25064373 10.1093/hmg/ddu328 PMC4170722

[26] Carter AR Sanderson E Hammerton G. Mendelian randomisation for mediation analysis: current methods and challenges for implementation. Eur J Epidemiol. 2021;36:465–78. 33961203 10.1007/s10654-021-00757-1 PMC8159796

[27] Papadimitriou N Dimou N Tsilidis KK. Physical activity and risks of breast and colorectal cancer: a Mendelian randomisation analysis. Nat Commun. 2020;11:597. 32001714 10.1038/s41467-020-14389-8 PMC6992637

[28] Hemani G Zheng J Elsworth B. The MR-Base platform supports systematic causal inference across the human phenome. Elife. 2018;7:e34408. 29846171 10.7554/eLife.34408 PMC5976434

[29] Staley JR Blackshaw J Kamat MA. PhenoScanner: a database of human genotype-phenotype associations. Bioinformatics. 2016;32:3207–9. 27318201 10.1093/bioinformatics/btw373 PMC5048068

[30] Bowden J Davey Smith G Burgess S. Mendelian randomization with invalid instruments: effect estimation and bias detection through Egger regression. Int J Epidemiol. 2015;44:512–25. 26050253 10.1093/ije/dyv080 PMC4469799

[31] Bowden J Davey Smith G Haycock PC. Consistent estimation in mendelian randomization with some invalid instruments using a weighted median estimator. Genet Epidemiol. 2016;40:304–14. 27061298 10.1002/gepi.21965 PMC4849733

[32] Burgess S Dudbridge F Thompson SG. Combining information on multiple instrumental variables in Mendelian randomization: comparison of allele score and summarized data methods. Stat Med. 2016;35:1880–906. 26661904 10.1002/sim.6835 PMC4832315

[33] Black LP Puskarich MA Henson M. Quantitative and qualitative assessments of cholesterol association with bacterial infection type in sepsis and septic shock. J Intensive Care Med. 2021;36:808–17. 32578468 10.1177/0885066620931473 PMC8061684

[34] Jang DH Jo YH Suh GJ. High cholesterol concentrations as well as low cholesterol concentrations are associated with mortality at 28 days in sepsis: a retrospective cohort study. Ann Palliat Med. 2021;10:10338–48. 34551576 10.21037/apm-21-1461

[35] Guirgis FW Black LP Rosenthal MD. LIPid intensive drug therapy for sepsis pilot (LIPIDS-P): phase I/II clinical trial protocol of lipid emulsion therapy for stabilising cholesterol levels in sepsis and septic shock. BMJ Open. 2019;9:e029348. 10.1136/bmjopen-2019-029348 PMC6756323 31537565

[36] Tanaka S Genève C Zappella N. Reconstituted high density lipoprotein therapy improves survival in mouse models of sepsis. Anesthesiology. 2020;132:825–38. 32101976 10.1097/ALN.0000000000003155

[37] De Nardo D Labzin LI Kono H. High-density lipoprotein mediates anti-inflammatory reprogramming of macrophages via the transcriptional regulator ATF3. Nat Immunol. 2014;15:152–60. 24317040 10.1038/ni.2784 PMC4009731

[38] Norata GD Catapano AL. Molecular mechanisms responsible for the antiinflammatory and protective effect of HDL on the endothelium. Vasc Health Risk Manag. 2005;1:119–29. 17315398 10.2147/vhrm.1.2.119.64083 PMC1993938

[39] Lekkou A Mouzaki A Siagris D. Serum lipid profile, cytokine production, and clinical outcome in patients with severe sepsis. J Crit Care. 2014;29:723–7. 24891152 10.1016/j.jcrc.2014.04.018

[40] Cirstea M Walley KR Russell JA. Decreased high-density lipoprotein cholesterol level is an early prognostic marker for organ dysfunction and death in patients with suspected sepsis. J Crit Care. 2017;38:289–94. 28013095 10.1016/j.jcrc.2016.11.041

[41] Walley KR Boyd JH Kong HJ. Low low-density lipoprotein levels are associated with, but do not causally contribute to, increased mortality in sepsis. Crit Care Med. 2019;47:463–6. 30394916 10.1097/CCM.0000000000003551

